# Priming neuronal regeneration: early adaptive mechanisms in zebrafish CNS injury

**DOI:** 10.3389/fnmol.2026.1807396

**Published:** 2026-04-16

**Authors:** Ignacio Babiloni Chust, Lucia Poggi, Michela Alessandra Denti, Andrea Pedroni

**Affiliations:** Department of Cellular, Computational and Integrative Biology, University of Trento, Trento, Italy

**Keywords:** calcium signaling, CNS regeneration, DAMP signaling, injury response, neuroinflammation, neuronal lesion, neurotransmitter plasticity, zebrafish

## Abstract

The zebrafish (*Danio rerio*) exhibits a remarkable capacity to regenerate the central nervous system following injury, restoring both tissue architecture and neural function. This review focuses on the earliest phases of the injury response, when conserved damage signals are first sensed, integrated, and translated into trajectories that bias tissue outcomes toward regeneration or persistent degeneration. We examine how early molecular cues, including damage-associated molecular patterns, inflammatory signals, calcium, and redox dynamics are differentially interpreted in zebrafish compared to mammals, leading to distinct cellular and tissue-level responses. Within this early signaling landscape, emerging evidence indicates that neuronal activity and neurotransmitter plasticity constitute a neuron-specific regulatory layer fundamental to the regulation of early injury responses and the initiation of regenerative programs. Rather than cataloging downstream regenerative mechanisms, we emphasize the importance of early temporal coordination of these injury-derived signals, which establishes permissive or non-permissive regulatory states. Overall, this review positions the zebrafish as a powerful vertebrate model for identifying general principles of early signal integration and temporal control that govern regenerative competence, providing a conceptual framework that may inform strategies to enhance repair in the injured mammalian CNS.

## 1 Introduction

The capacity to regenerate damaged tissues is an ancient biological trait, yet it is unevenly retained across the animal kingdom (Bely, 2010; Brockes and Kumar, 2008; Elchaninov et al., 2021; Poss, 2010; Yun, 2015). While many invertebrates exhibit extraordinary regenerative competence, including the ability to rebuild entire organs or body plans (Kostyuchenko and Kozin, 2021; Reddien, 2018; Reddy et al., 2019), most vertebrates display restricted reparative potential. In mammals, tissue injury is frequently resolved through scar formation rather than complete regeneration (Harty et al., 2003; Iismaa et al., 2018; Jiang and Rinkevich, 2020; Yannas and Tzeranis, 2021), often resulting in permanent loss of structure and function, particularly within the central nervous system (CNS) (Blackshaw, 2022; Varadarajan et al., 2022). In contrast, varying degrees of regenerative capacity are retained across several non-mammalian vertebrate lineages, including restoration of the CNS (Joven et al., 2019; Zupanc and Sîrbulescu, 2013), suggesting that regenerative programs were not lost during vertebrate evolution but rather became differentially regulated across the phylum (Bely, 2010; Elchaninov et al., 2021).

Current evidence indicates that this divergence may reflect an evolutionary trade-off associated with increasing immune complexity, vascular specialization, and long-term circuit stability (Harty et al., 2003; Jaźwińska and Sallin, 2016; Jiang and Rinkevich, 2020). Selective pressures appear to have favored rapid damage containment and infection control over extensive cellular plasticity and structural remodeling (Bely, 2010; Blackshaw, 2022; Elchaninov et al., 2021; Poss, 2010).

In this context, zebrafish has emerged as a leading vertebrate model for dissecting the mechanisms underlying successful CNS regeneration. Zebrafish shares extensive genetic, molecular, and physiological homology with mammals, yet it maintains robust regenerative responses in several adult tissues, including the brain, retina, and spinal cord (Gemberling et al., 2013; Ghosh and Hui, 2016; Kizil et al., 2012; Major and Poss, 2007). This combination of evolutionary proximity and regenerative competence positions zebrafish as an exceptionally powerful system for identifying conserved regulatory mechanisms that enable successful neural repair. It should be noted that the experimental studies discussed in this review include work performed at different developmental stages, ranging from larval to juvenile and adult zebrafish. Despite these differences, current evidence suggests that several of the molecular pathways involved in early injury sensing and signal integration are shared across developmental stages and remain active throughout adult life (Alper and Dorsky, 2022; Becker and Becker, 2008; Gemberling et al., 2013; Kizil et al., 2012; Wan and Goldman, 2016).

**FIGURE 1:**
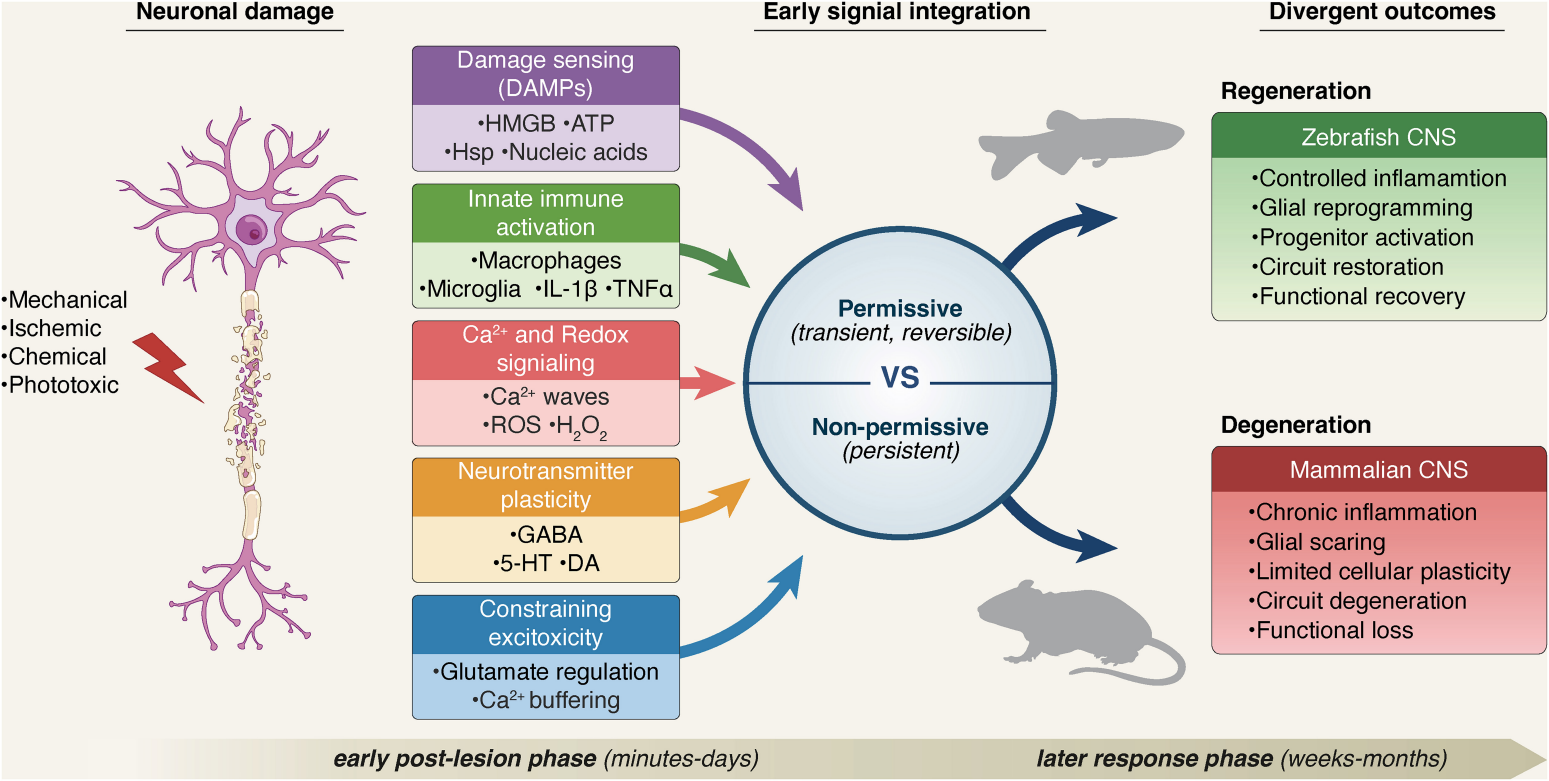
Early signal integration determines divergent CNS injury outcomes in zebrafish and mammals. Following CNS injury, conserved damage-associated signals, including DAMP release, immune activation, calcium and redox dynamics, neurotransmitter plasticity, and excitotoxic stress control, are rapidly sensed and integrated across multiple cellular layers during the early post-lesion phase. In zebrafish, tight temporal coordination and resolution of these early responses promote a permissive integrative state, enabling glial reprogramming, progenitor activation, circuit stabilization, and functional recovery. In contrast, in the mammalian CNS, altered timing and persistence of comparable signals bias integration toward non-permissive states characterized by chronic inflammation, glial scarring, circuit destabilization, and progressive degeneration.

Most existing reviews of zebrafish CNS regeneration focus exhaustively on downstream injury processes such as glial proliferation, neurogenesis, axonal regrowth, and circuit reconstruction (Akram et al., 2022; Gallo and Deneen, 2014; Magdesian et al., 2016; Zambusi and Ninkovic, 2020). While these processes are essential for tissue restoration, accumulating evidence indicates that early responses set boundary conditions that constrain later regenerative trajectories, beginning within minutes after injury and extending throughout the first few days of the post-lesion response (Barreiro-Iglesias et al., 2015; Ghaddar et al., 2021; Kuscha et al., 2012a; Pedroni et al., 2024; Reimer et al., 2013; Sifuentes et al., 2016). During this early temporal window, conserved damage signals are detected, interpreted, and integrated by resident neural, glial, and immune cells. It is at this stage that the injured tissue commits either to a transient adaptive response permissive for regeneration, or to a maladaptive trajectory characterized by chronic inflammation, glial scarring, tissue degeneration, and persistent functional loss. Accordingly, this review focuses specifically on the earliest phases of CNS injury response in zebrafish. We examine how damage-associated molecular patterns (DAMPs), ionic fluxes, calcium waves, and reactive oxygen species (ROS) initiate rapid molecular cascades that prime regenerative competence. We then discuss how transient immune activation and early neurochemical reorganization converge to stabilize neural circuits while establishing and maintaining permissive conditions for coordinated regenerative programs (Figure 1).

Together, these observations motivate a shift in perspective, framing zebrafish CNS regeneration not as a delayed reparative process, but as the cumulative outcome of early signal integration and temporally constrained decision-making following injury. Within this framework, this review highlights regulatory principles that distinguish regenerative from non-regenerative repair trajectories and may inform future translational strategies aimed at enhancing repair in the mammalian CNS.

## 2 Damage sensing and early signal integration in CNS regeneration

A growing body of work identifies the earliest phase of CNS injury sensing as a critical molecular crossroads, at which conserved damage signals are detected by resident neural, glial, endothelial, and immune cells and translated into divergent biological outcomes across vertebrates. How these signals are decoded and integrated within this early temporal window is likely decisive in determining whether injured tissue commits to a transient, regeneration-permissive state or instead enters a maladaptive trajectory characterized by chronic inflammation, cellular silencing, tissue degeneration, scar formation, and functional loss.

Regardless of whether the insult is mechanical, ischemic, chemical, or phototoxic, cellular stress or loss of cellular integrity leads to the release of DAMPs. Molecules such as high-mobility group box 1 (HMGB1), purine metabolites including adenosine triphosphate (ATP), heat shock proteins, and nucleic acids normally perform intracellular homeostatic or metabolic functions; however, once released into the extracellular milieu, they are converted into potent alarmins (Frank et al., 2015; Kunze et al., 2023). In this context, DAMPs act as primary ligands for a conserved set of pattern-recognition receptors (PRRs) expressed by multiple resident cell types within the CNS. These include Toll-like receptors (TLRs), purinergic receptors (P2X/P2Y), and the receptor for advanced glycation end products (RAGE) (Chen et al., 2025; Zhang et al., 2023). Activation of these receptors alerts the innate immune system and initiates a form of inflammation commonly referred to as sterile inflammation (Chen et al., 2025; Gong et al., 2020; Liesz et al., 2015; Lin et al., 2025).

In the zebrafish CNS, DAMP sensing is tightly linked to the activation of molecular programs that support cell-cycle re-entry and neuroregeneration. For example, the release of extracellular RNA from damaged and dying cells, followed by TLR3 activation, is required for the recruitment of embryonically derived, dormant ependymo-radial glial cells during spinal cord regeneration (Vandestadt et al., 2021). Similarly, HMGB1 signaling is induced in neurons and blood vessels, where it has been implicated in the stimulation of both neurogenesis and angiogenesis during spinal cord repair (Fang et al., 2014). In the retina, ATP released during photoreceptor degeneration triggers Müller glial reprogramming through purinergic signaling (Brandli et al., 2021). Collectively, these examples illustrate how injury sensing in the zebrafish CNS is translated into a permissive state that supports subsequent neurogenesis and tissue repair.

By contrast, in the mammalian CNS, the same DAMPs are generally interpreted as danger signals that reinforce inflammatory and gliotic programs, thereby promoting neurodegeneration rather than regeneration (Di Virgilio et al., 2009; Fan et al., 2020; Leiba et al., 2023; Puthussery and Fletcher, 2009). Consistent with this framework, therapeutic strategies aimed at modulating DAMP-sensing receptors have been proposed as a means to counteract sterile inflammation and limit the progression of neurodegenerative diseases (Ma et al., 2024).

Taken together, these observations indicate that although DAMPs and their receptors are highly conserved across vertebrates, their downstream interpretation and integration constitute a critical divergence point between regenerative and non-regenerative CNS responses. This early regulatory context constrains how subsequent immune, glial, and neuronal responses are coordinated and ultimately resolved.

Elucidating whether this divergence arises from differences in cell identity, receptor coupling and signaling dynamics, or epigenetic regulation is essential. Deciphering these mechanisms could enable the reprogramming of the default defensive scarring response toward cellular plasticity and active regeneration.

## 3 Inflammation timing and resolution as determinants of regenerative outcomes

Beyond initial DAMP sensing, immune activation, and more critically the duration and resolution of inflammatory states, have emerged as a decisive early checkpoint shaping CNS injury outcomes, steering tissues toward regeneration or alternatively toward scarring and degeneration (Bludau et al., 2024; García-García et al., 2024; Zhang et al., 2023). In zebrafish, the inflammatory phase is not only rapid and self-limiting (Moritz et al., 2015), but is also promptly converted into a permissive signal that promotes glial reprogramming, progenitor activation, and neurogenesis (Bludau et al., 2024; García-García et al., 2024; Iribarne, 2021; Iribarne and Hyde, 2022; Kizil, 2018; Nagashima and Hitchcock, 2021). Accordingly, key pro-inflammatory signals, including IL-1β and TNF-α, as well as immune cell populations such as macrophages and resident microglia, act not only as immune effectors but also as instructive cues that actively support CNS regeneration (Nagashima and Hitchcock, 2021; Shimizu et al., 2021; Tsarouchas et al., 2018; Var and Byrd-Jacobs, 2020).

Consistent with this instructive role, pharmacological suppression of inflammation or depletion of specific immune mediators impairs regenerative capacity in the zebrafish CNS, underscoring that acute inflammation is necessary, although not sufficient, for successful repair (Bludau et al., 2024; García-García et al., 2024; Iribarne and Hyde, 2022; Palsamy et al., 2023; Var and Byrd-Jacobs, 2020).

In the mammalian CNS, by contrast, immune activation more frequently evolves into sustained, chronic inflammation, characterized by persistent cytokine signaling, prolonged glial reactivity, continued immune cell presence, secondary cell death, extracellular matrix deposition, and scar formation that collectively limit regenerative potential (Fan et al., 2020; Zhang et al., 2023). These differences have prompted increasing efforts to counteract or accelerate the resolution of inflammation as a prerequisite for enabling regenerative responses in the injured mammalian CNS.

Notably, recent work has shown that photoreceptor regeneration can proceed normally following light-induced retinal damage in zebrafish mutants lacking microglia (Song et al., 2024), highlighting the complexity and context dependency of immune contributions to regeneration. Whether such outcomes reflect damage-specific immune requirements, compensatory mechanisms, or priming of the tissue environment by other cell types remains to be determined.

## 4 Calcium and redox dynamics in early CNS injury responses

Among the earliest CNS injury-induced signals are rapid calcium influxes (Ca^2+^ waves) and the production of reactive oxygen species (ROS), particularly hydrogen peroxide (H_2_O_2_), which itself functions as a DAMP. Together, these signals act as fast, spatially restricted integrators that translate tissue disruption into coordinated cellular responses before the full engagement of inflammatory cascades (Liu et al., 2021; Weber, 2012; Yoo et al., 2012). Their strong evolutionary conservation supports the view that Ca^2+^ and redox signaling represent ancient mechanisms linking tissue damage to neuronal, glial, and innate immune activation (Khaitin, 2021; Niethammer et al., 2009; Siauciunaite et al., 2019; Weber, 2012).

In zebrafish models, injury-induced calcium waves and transient ROS production act as early intermediates linking tissue damage to regenerative responses (Anand et al., 2021; Chen et al., 2019; Romero et al., 2018; Sipka et al., 2021). However, how calcium signaling, ROS dynamics, and oxidative stress are integrated across neurons, radial glia, and microglia in the injured zebrafish CNS remains incompletely understood.

Importantly, regenerative contexts appear to rely on the tight spatial and temporal confinement of ROS signaling, allowing these molecules to function as instructive second messengers rather than cytotoxic agents (Gauron et al., 2013; Narra et al., 2023). By contrast, in the mammalian CNS, prolonged calcium dysregulation and sustained ROS production drive oxidative stress, amplify inflammatory signaling, promote cell death, and stabilize non-permissive tissue states (Sanabria-Castro et al., 2024).

Consistent with this view, antioxidant-based strategies have been widely explored in mammalian models to limit neurodegeneration and scar formation (Iakovou and Kourti, 2022; Patel, 2016). However, emerging evidence suggests that ROS signaling can also exert context-dependent instructive roles. Notably, modulation of ROS dynamics has recently been shown to promote neuroepithelial progenitor reprogramming in the injured cerebellum, where spatially controlled ROS signaling stimulates adaptive glial responses and tissue repair (Pakula et al., 2025).

Together, these findings challenge the view of ROS and inflammation as purely pathological processes and support instead a model in which they form a tightly coupled, temporally structured signaling module. While ROS and inflammation are often described as mutually reinforcing processes, fueling a longstanding debate regarding regenerative causality, effective regenerative competence may depend less on suppressing these signals than on precisely controlling their timing, magnitude, and resolution.

The ability to initiate and subsequently terminate this early oxidative–inflammatory loop therefore emerges as a critical checkpoint that biases CNS injury responses toward regenerative rather than degenerative trajectories. In doing so, it establishes a permissive physiological landscape upon which additional activity-dependent regulatory layers can be engaged.

Crucially, calcium signaling does not operate solely as an upstream modulator of inflammation. Through its central role in controlling neuronal excitability, synaptic transmission, and neurotransmitter release, Ca^2+^ dynamics occupy a privileged position at the interface between molecular injury sensing and neuronal function. By directly coupling tissue damage to changes in network activity and transmitter output, calcium provides a mechanistic bridge through which early injury cues can be translated into neurochemical reorganization, positioning neuronal signaling as an active regulatory layer in early CNS regeneration.

## 5 Neurotransmitter plasticity as a neuron-specific regulatory layer in CNS regeneration

Unlike other regenerating tissues, the nervous system possesses an intrinsic, activity-dependent signaling layer based on neurotransmitter release, that enables neurons to directly participate in early injury integration and in the regulation of regenerative processes.

Neurotransmitter plasticity, including activity-dependent transmitter switching, represents a conserved and widespread regulatory mechanism across vertebrate nervous systems, contributing to circuit adaptation, homeostatic control, and behavioral flexibility (Dulcis et al., 2013; Li et al., 2020; Meng et al., 2018; Spitzer, 2015, 2017).

The adult zebrafish exhibits a remarkable degree of neurotransmitter plasticity even under physiological conditions, reflecting the capacity of mature neurons to adjust their transmitter identity and synaptic interactions in response to activity-dependent and systemic demands (Bertels et al., 2022; Bertuzzi et al., 2018; Pedroni and Ampatzis, 2019). This intrinsic flexibility provides a permissive substrate upon which injury-induced neurochemical reorganization can be rapidly deployed.

Following CNS injury, the neurochemical equilibrium is rapidly disrupted, necessitating early compensatory mechanisms that stabilize excitability and protect circuit integrity. Within hours, a precisely orchestrated sequence of changes in neurotransmitter synthesis, release, and receptor expression is initiated. These early adaptations serve two tightly coupled functions: they contribute to the rapid re-establishment of excitatory–inhibitory balance, thereby limiting secondary damage and proximal degeneration, and they provide instructive cues that promote glial activation, progenitor proliferation, and neuronal differentiation, thereby initiating regenerative cascade.

## 6 Transient GABAergic disinhibition as permissive signal for regenerative programs

A prominent example of lesion-induced neurochemical adaptation in zebrafish is the transient reduction of GABAergic signaling observed across multiple regenerative contexts. GABAergic inhibition plays a central role in maintaining network excitability and homeostasis in vertebrates, primarily through the action of GABA_A_ receptors (Smart and Stephenson, 2019). These receptors mediate two complementary forms of inhibition: phasic inhibition at synaptic sites through transient GABA release, and tonic inhibition mediated by extrasynaptic receptors persistently activated by ambient or spillover GABA, thereby setting the excitability threshold of neuronal populations (Lee et al., 2010; Mortensen et al., 2012).

At the onset of the injury response, a controlled and spatially restricted downregulation of GABAergic tone transiently relieves this inhibitory constraint, creating permissive conditions for progenitor activation and network remodeling. In the adult zebrafish spinal cord, non-synaptic GABAergic transmission regulates neural stem and progenitor cell (NSPC) activity through a dynamic interplay with nicotinic acetylcholine receptors (nAChRs). Following spinal injury, pharmacological inhibition of GABA_A_ receptors combined with nAChR activation enhances neurogenesis and supports motor function recovery (Chang et al., 2021).

A comparable mechanism operates in the zebrafish retina, where suppression of GABA signaling is sufficient to induce Müller glia dedifferentiation and proliferation even in the absence of overt tissue damage, whereas sustained GABA_A_ receptor activation inhibits regeneration in injured retinas (Kent et al., 2021; Rao et al., 2017). Together, these findings indicate that transient reduction of GABAergic tone functions as an early permissive signal that gates access to regenerative programs. By modulating network excitability, this mechanism establishes conditions that are required for glial activation and progenitor recruitment during the initial phases of regeneration.

## 7 Monoaminergic control of progenitor activation and circuit reorganization during early CNS regeneration

Monoaminergic systems, particularly serotonin and dopamine, are well-established modulators of zebrafish CNS regeneration, influencing progenitor activation, axonal regrowth, and circuit reorganization after injury. Following spinal cord injury, both neurotransmitters promote motor neuron regeneration through partially independent mechanisms (Barreiro-Iglesias et al., 2015; Kuscha et al., 2012a,b; Pérez et al., 2013; Reimer et al., 2013), with several of these studies showing that manipulation of monoaminergic pathways during the first days after lesion influences regenerative outcomes at later stages of recovery.

Following spinal cord injury, serotonin stimulates pMN-like ependymo-radial glia (ERGs) to reinitiate adult neurogenesis by redeploying a developmental pMN proliferative program (Barreiro-Iglesias et al., 2015). However, adult pMN-like ERGs preferentially express htr1aa but not htr1ab, indicating a shift in receptor subtype utilization between development and regeneration. In addition, serotonin released from descending axons rostral to the lesion is necessary and sufficient to promote proliferation of pMN-like progenitors, whereas caudal to the lesion exogenous serotonin enhances progenitor-driven motor neuron production, highlighting the importance of an early injury-induced perilesional neurochemical configuration (Barreiro-Iglesias et al., 2015; Kuscha et al., 2012a). Importantly, serotonergic stimulation alone is insufficient to induce progenitor proliferation in the absence of injury, indicating that monoaminergic cues act only after permissive states have been established.

In addition to progenitor regulation, monoaminergic signaling also contributes to circuit-level reorganization after injury. A distinct population of injury-induced intraspinal serotonergic neurons (ISNs) promotes axonal regrowth of long-projecting glutamatergic interneurons via 5-HT1B receptor activation, supporting subsequent circuit remodeling and functional locomotor recovery; disruption of this pathway impairs restoration of motor function (Huang et al., 2021).

Dopaminergic signaling is similarly redeployed during adult spinal cord regeneration, recapitulating developmental brain–spinal communication pathways that modulate progenitor output through D4a receptor activation and Hedgehog signaling modulation (Reimer et al., 2013, 2009). During regeneration, supraspinal dopaminergic control is reinstated: brain-derived dopamine enhances motor neuron production and alters progenitor receptor profiles both rostral and caudal to the lesion.

Together, these findings highlight how monoaminergic systems operate as neuron-specific regulators that act within an early regenerative phase following injury, targeting both progenitor populations and nascent circuits once injury-induced permissive states have been established. Consistent with a tightly timed and tightly regulated regenerative program, spinal regeneration in zebrafish involves an initial expansion of neuronal output, including serotonergic and motor neuron populations, followed by a selective pruning phase that eliminates excess cells and refines circuit architecture. This sequence indicates that monoaminergic signaling contributes not only to the early amplification of regenerative output, but also to its subsequent structuring and refinement, enabling the recovery of functional neural circuits.

By re-engaging conserved developmental signaling logics, monoamines thus provide a mechanistic link between early injury responses and the deployment and maturation of regenerative neurogenesis and circuit reorganization in the adult zebrafish CNS. The extent to which differences in the engagement or temporal coordination of comparable monoaminergic pathways contribute to the limited regenerative capacity of the mammalian CNS remains to be determined.

## 8 Homeostatic control of excitatory signaling during early regeneration

Glutamatergic signaling plays a critical and context-dependent role during the early phases of CNS regeneration, where its precise temporal and spatial regulation is essential for regenerative success. While glutamate is indispensable for activity-dependent synaptogenesis and circuit formation, excessive extracellular accumulation following injury can induce excitotoxicity and exacerbate tissue damage.

In the zebrafish retina, glutamatergic signaling indirectly regulates MG activation. Under physiological conditions, glutamate released by photoreceptors stimulates GABAergic horizontal cells, maintaining ambient inhibition and MG quiescence. Following photoreceptor degeneration, loss of glutamatergic drive reduces GABA release, creating a permissive decrease in inhibitory tone that enables MG dedifferentiation. Consistently, pharmacological inhibition of AMPA receptors is sufficient to trigger MG proliferation in the absence of injury, whereas their activation suppresses regeneration (Rao et al., 2017).

In the spinal cord, injury induces an immediate transient increase in glutamatergic input and intracellular calcium in motoneurons, accompanied by upregulation of calretinin and connexin 35/36 expression. This response enhances gap junction–mediated coupling, facilitating the redistribution of excess calcium to buffer cytotoxic load and preserve network integrity. Disruption of this coupling increases early neuronal death and delays subsequent lesion bridging, revealing a glutamate-dependent, connexin-mediated neuroprotective mechanism that supports early injury responses and regeneration (Pedroni et al., 2024).

Together, these observations indicate that regenerative success depends not on suppressing glutamatergic signaling, but on its precise temporal and spatial regulation, allowing activity-dependent plasticity to be preserved while limiting excitotoxic stress during the early post-injury phase.

More broadly, these findings position neurotransmitter plasticity as an integrative component of the early CNS injury response in zebrafish. Injury-induced neurochemical changes converge on restoring ionic and excitability balance and on activating glial and progenitor responses that initiate neurogenesis. These shifts are tightly regulated in space and time, generating transient permissive states that are subsequently resolved as regeneration progresses. Rather than acting solely as synaptic messengers, neurotransmitters function as rapid contextual cues that link neuronal activity to early injury sensing and regenerative responses. Understanding how this neurochemical layer intersects with early damage signals and inflammatory dynamics remains an important and relatively underexplored frontier in zebrafish CNS regeneration.

## 9 Discussion

Viewed collectively, the mechanisms discussed in this review converge on a unifying concept: regenerative outcomes in the zebrafish CNS are determined not by the activation of regeneration-specific pathways *per se*, but by the early coordination, timing, and resolution of conserved injury responses. Rapid damage sensing, transient immune activation, tightly controlled calcium and redox signaling, and early neurochemical plasticity operate within a defined early post-lesional temporal window to bias injured tissue toward a permissive regenerative state rather than toward chronic inflammation, scarring, and functional loss.

The regenerative capacity of the zebrafish CNS thus emerges as a systems-level property of early signal integration. Rather than arising from isolated downstream processes such as neurogenesis or axonal regrowth, successful repair reflects the ability of injured tissue to interpret damage-derived cues in a coordinated and reversible manner, aligning molecular, cellular, and circuit-level responses toward restoration.

From an evolutionary perspective, this organization reflects an ancestral regenerative strategy that has been progressively constrained during vertebrate evolution (Bely, 2010; Brockes and Kumar, 2008; Elchaninov et al., 2021; Iismaa et al., 2018; Tanaka and Reddien, 2011). Increasing anatomical specialization, immune complexity, metabolic efficiency, and long-term circuit stability likely imposed selective pressures favoring rapid damage containment and preservation of established network architecture over extensive cellular plasticity and structural remodeling (Harty et al., 2003; Jaźwińska and Sallin, 2016; Jiang and Rinkevich, 2020). Importantly, comparative evidence indicates that the molecular substrates underlying regeneration, including stress signaling pathways, immune mediators, calcium and redox signaling, and neurochemical plasticity, remain largely conserved across vertebrates. The primary divergence appears to reside not in genetic content, but in the regulatory context governing the timing, magnitude, and reversibility of pathway activation (Becker and Becker, 2008; Poss, 2010; Tanaka and Reddien, 2011; Wan and Goldman, 2016). In this light, zebrafish regeneration should not be viewed as an exception, but as a permissive and reversible regulatory state in which developmental and repair programs remain accessible throughout adulthood.

This framing carries direct translational implications. Rather than attempting to reproduce zebrafish regeneration wholesale in mammals, a more viable strategy involves selectively re-engaging discrete components of latent plasticity under tightly controlled conditions (Arjmand et al., 2020; Balzamino et al., 2025; Ceci et al., 2018; Gemberling et al., 2013). Recent preclinical studies in mammalian systems provide emerging support for this approach: transient immune modulation, controlled induction of glial stem-like states, targeted epigenetic remodeling, and metabolic reprogramming have each demonstrated that aspects of regenerative competence can be partially reinstated following CNS injury (Bonosi et al., 2022; Kvistad et al., 2024; Varadarajan et al., 2022; Wan and Ding, 2023; Wang et al., 2021; Yang et al., 2020). Collectively, these efforts suggest that successful repair depends less on single molecular interventions than on restoring temporal coordination and systemic coherence within the injured tissue, closely paralleling the early decision logic observed in zebrafish regeneration.

At the same time, translating regenerative mechanisms from zebrafish to the adult mammalian CNS raises important ethical and conceptual challenges. Inducing extensive cellular plasticity in the mature brain carries inherent risks, including uncontrolled proliferation, tumor formation, ectopic differentiation, aberrant circuit integration, and long-term cognitive or behavioral impairment (Fortin et al., 2016; Kvistad et al., 2024; Yadirgi and Marino, 2009; Ying et al., 2023). These risks underscore the need for precise spatiotemporal control of plasticity programs, rigorous regulation of cell fate decisions, and long-term functional assessment.

Conceptually, findings from zebrafish regeneration challenge the long-standing assumption that adult neuroplasticity in mammals is intrinsically limited or irreversibly lost (Case and Tessier-Lavigne, 2005; Olson, 1997; Varadarajan et al., 2022). Instead, regeneration may be more accurately understood as an evolutionarily conserved but differentially accessible process, constrained by species-specific immune dynamics, epigenetic regulation, and tissue architecture. In zebrafish, permissive regulatory environments allow injury-induced programs to unfold productively and resolve appropriately; in mammals, similar pathways are often prematurely silenced, restricted to narrow contexts, or activated in ways that stabilize non-permissive tissue states (Bely, 2010; Brockes and Kumar, 2008; Elchaninov et al., 2021; Iismaa et al., 2018; Tanaka and Reddien, 2011). Accordingly, future translational strategies must focus on identifying the thresholds beyond which regeneration becomes destabilizing rather than restorative.

In conclusion, zebrafish CNS regeneration demonstrates that functional recovery is achievable when adaptive responses are integrated across biological scales and constrained within appropriate temporal boundaries. Comparative analysis across species refines our understanding of vertebrate neurobiology, revealing that the genetic potential for CNS repair in mammals is not absent, but largely silenced. Deciphering the regulatory logic that allows zebrafish to access this latent potential provides a principled framework for restoring limited, yet meaningful, regenerative capacity in the injured human CNS.
